# Supplementary material for: Nano-Mod-Amp reveals RNA sequence, structural and cell type specific features of pseudouridylation by PUS7
Source: bioRxiv. 2025 Oct 31:2025.10.30.685621. Preprint. [Version 1] doi: 10.1101/2025.10.30.685621 (PMC12636312; doi:10.1101/2025.10.30.685621)
Supplement: Supplement 1 — Supplemental Figure 1: Nanopore direct RNA sequencing benchmarking and calibration standards. Related to Figure 1. (A) Schematic of UNUAR concatemer in unmodified and fully-pseudouridylated states. Each of the 8 UNUAR motifs are separated by a 14-nucleotide spacer. A polyA tail is appended to the end of the sequence to allow for integration in Nanopore direct RNA sequencing pipelines. (B) Genome browser style views for UNUAR concatemer at each of the UNUAR motifs. Unmodified template on top, fully pseudouridylated template on bottom. Nucleotide frequency is shown on the y-axis. Color-coded by nucleotide: blue, C; red, U. The pseudouridine is centered in the window. (C) U-to-C mismatch rates across the UNUAR concatemer for the 8 U-Ψ mixes sequenced. Positions follow the order of the motif in A. Non-U positions are shown as having a mismatch rate of 0. (D) Calibration curves for each of the UNUAR motifs. UNΨAR concentration is shown on the x-axis, observed U-to-C mismatch rate in direct RNA sequencing is shown on the y-axis. Line of best fit was determined through a linear model and fitted to each motif individually. (E) Uncorrected U-to-C mismatch rate and corrected Ψ stoichiometry in WT and KD of PUS7-dependent sites identified from direct RNA sequencing in at least two out of three replicates, with coverage > 10 and mismatch rate > 15% in a UNUAR motif. Corrected values determined from average U-to-C mismatch rate and UNUAR standard calibration curves. Sites are labeled with genomic location and motif containing the pseudouridine. (F) Distribution of PUS7-dependent pseudouridine identified in direct RNA sequencing with > 15% U-to-C mismatch rate across transcriptome regions and RNA type in comparison to background uridine distribution. All sites were observed in two out of three biological replicates and had > 10 reads of coverage. Supplemental Figure 2: Comparison of deletion rates obtained by Nanopore compared to Illumina amplicon sequencing. Related to Figure 2. [file media-1.pdf]

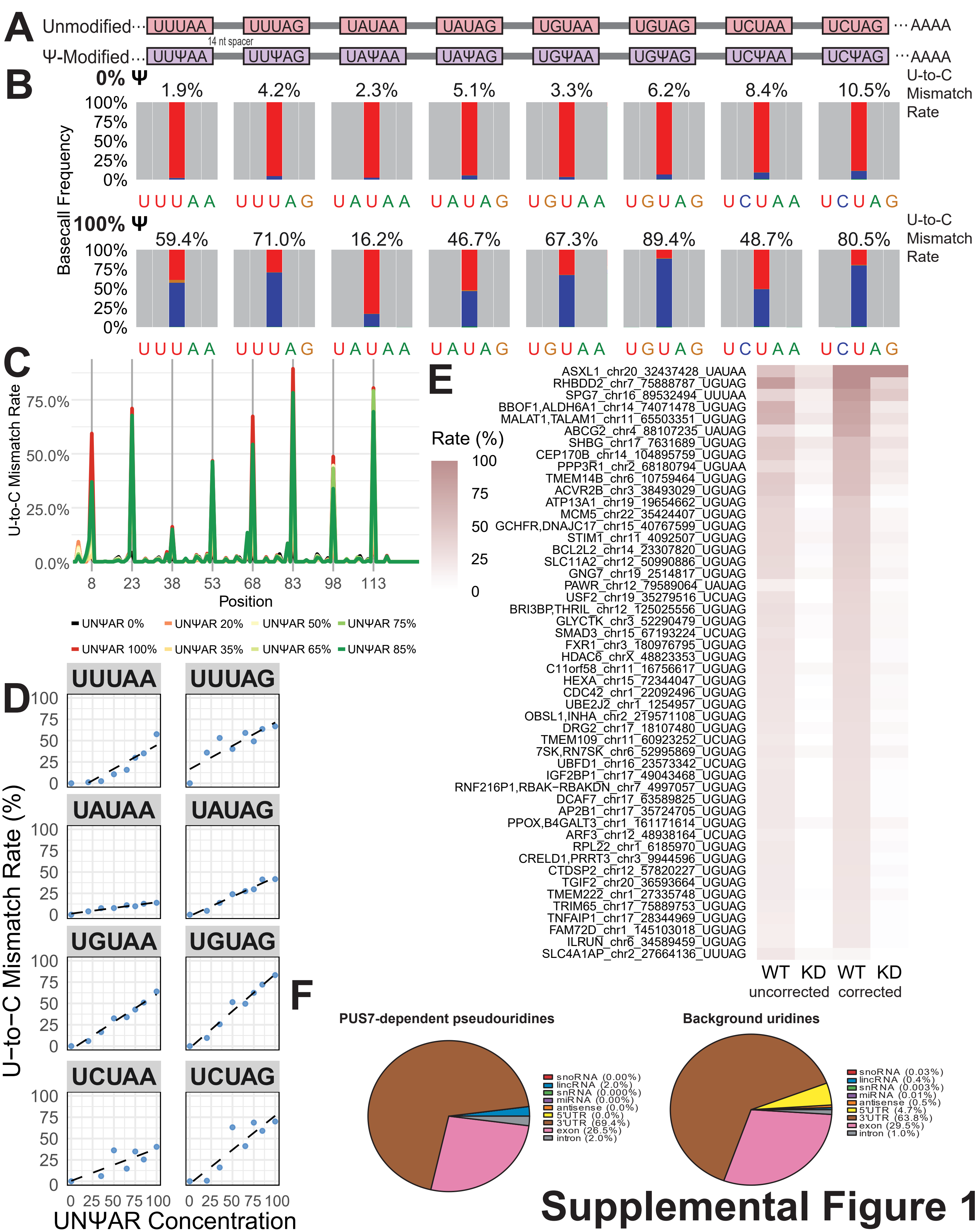

Supplemental Figure 1

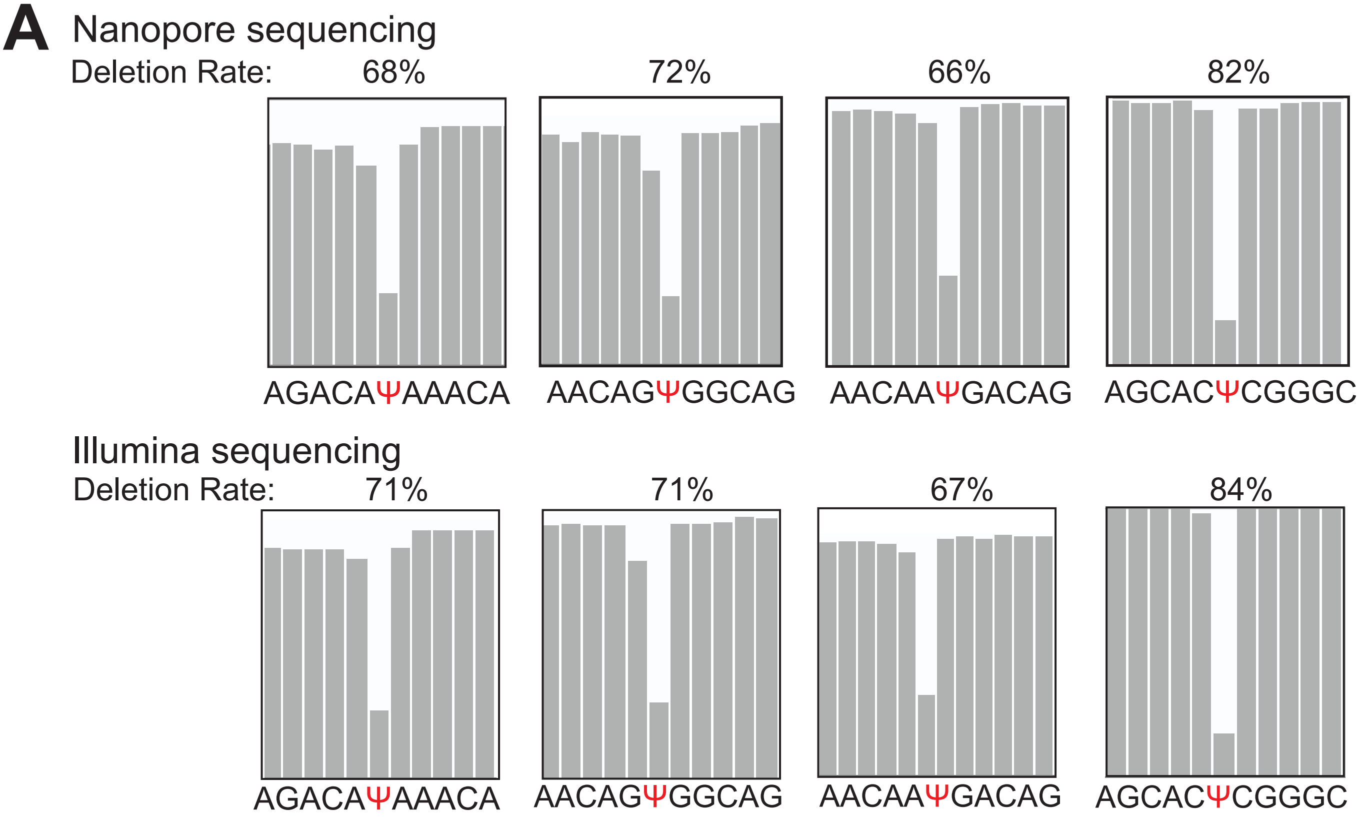

Supplemental Figure 2

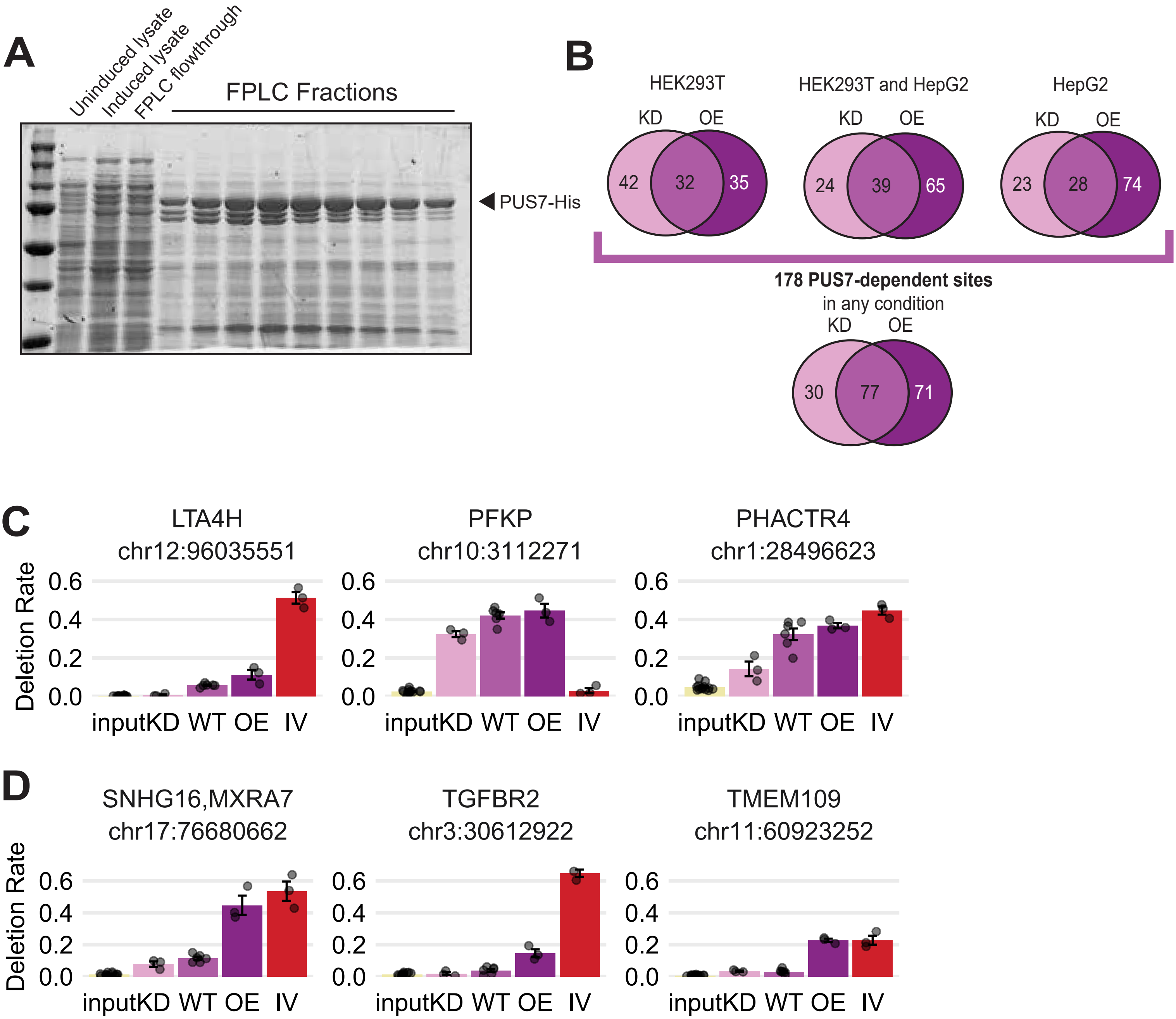

Supplemental Figure 3

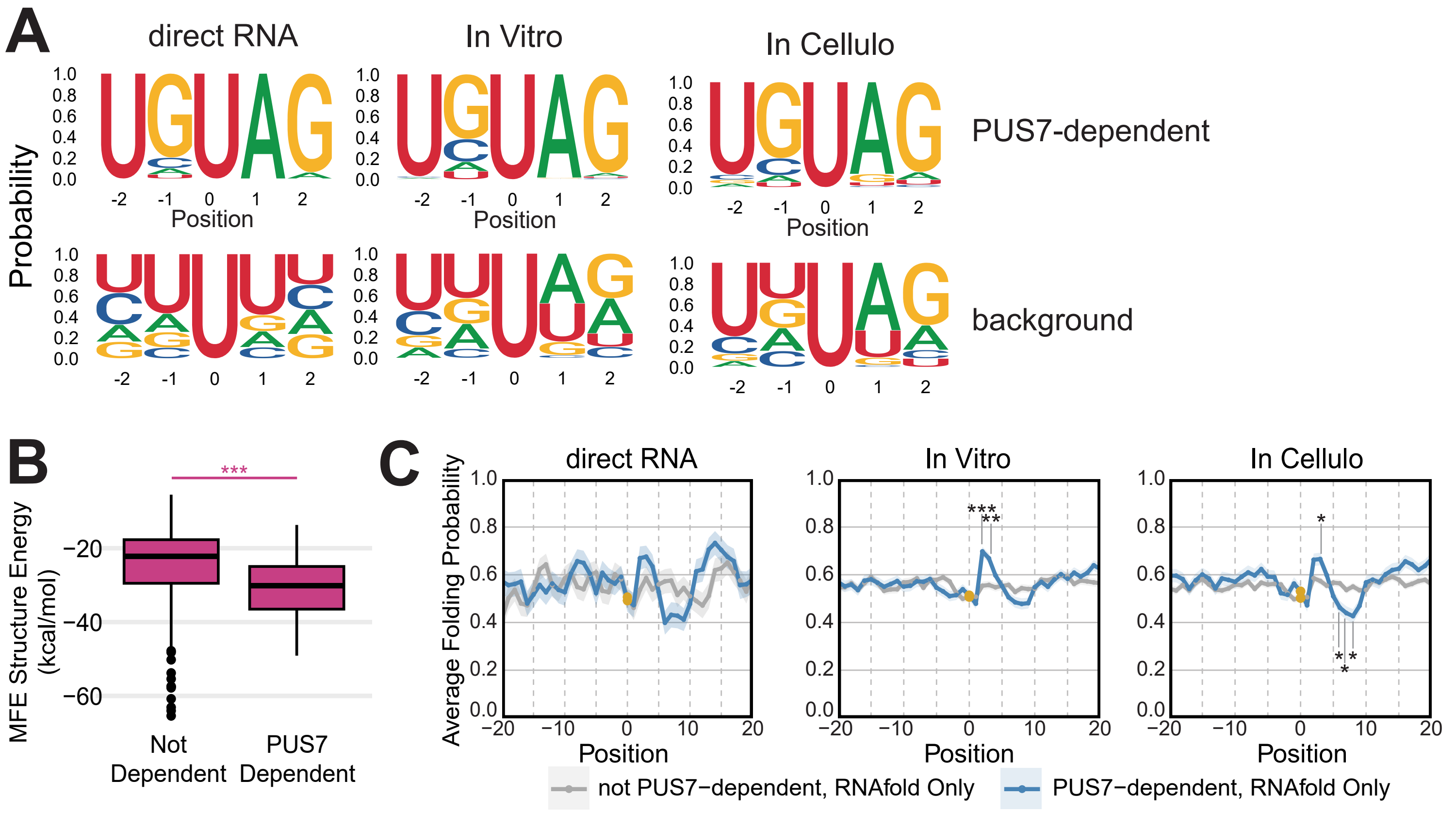

Supplemental Figure 4

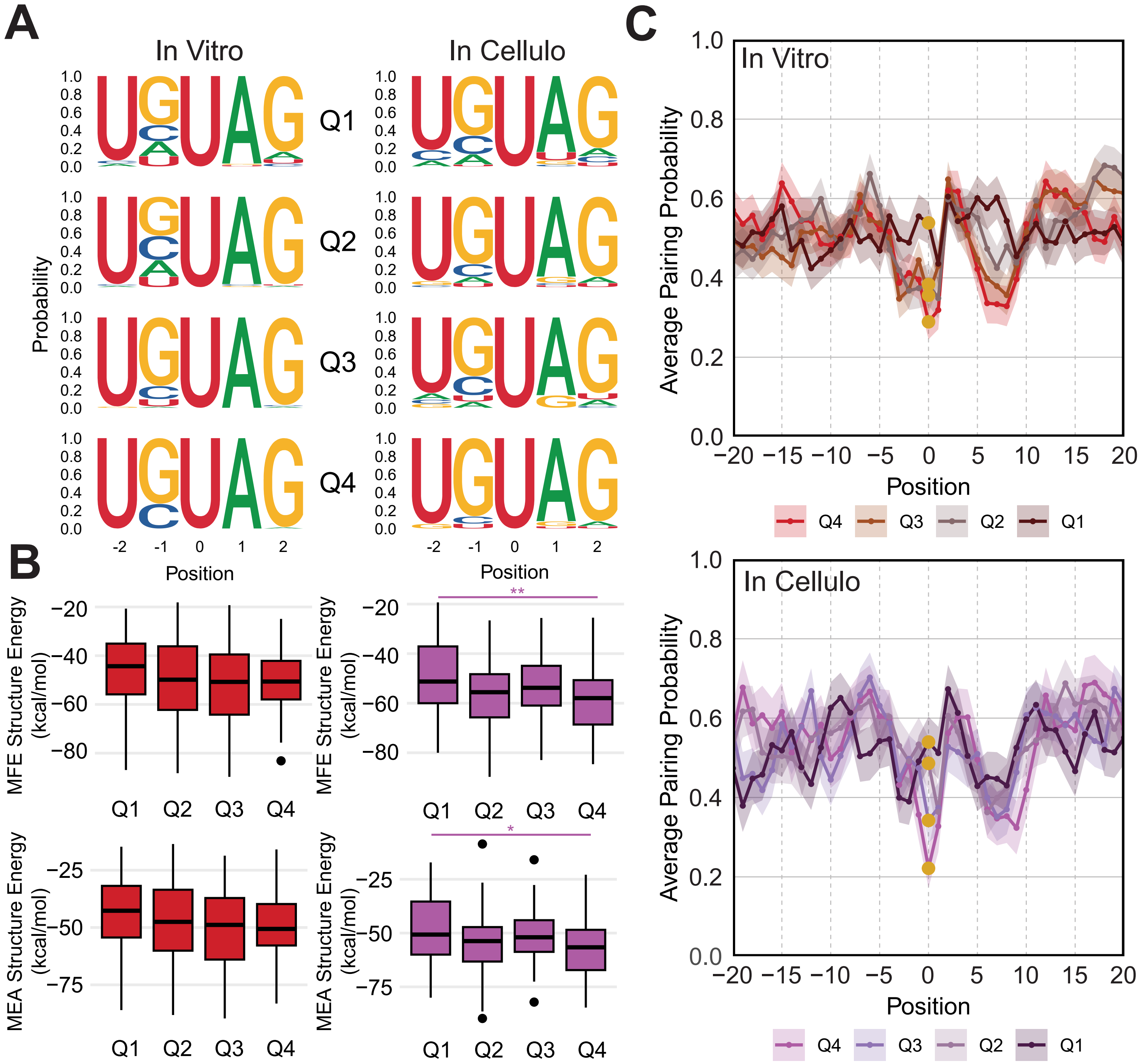

Supplemental Figure 5

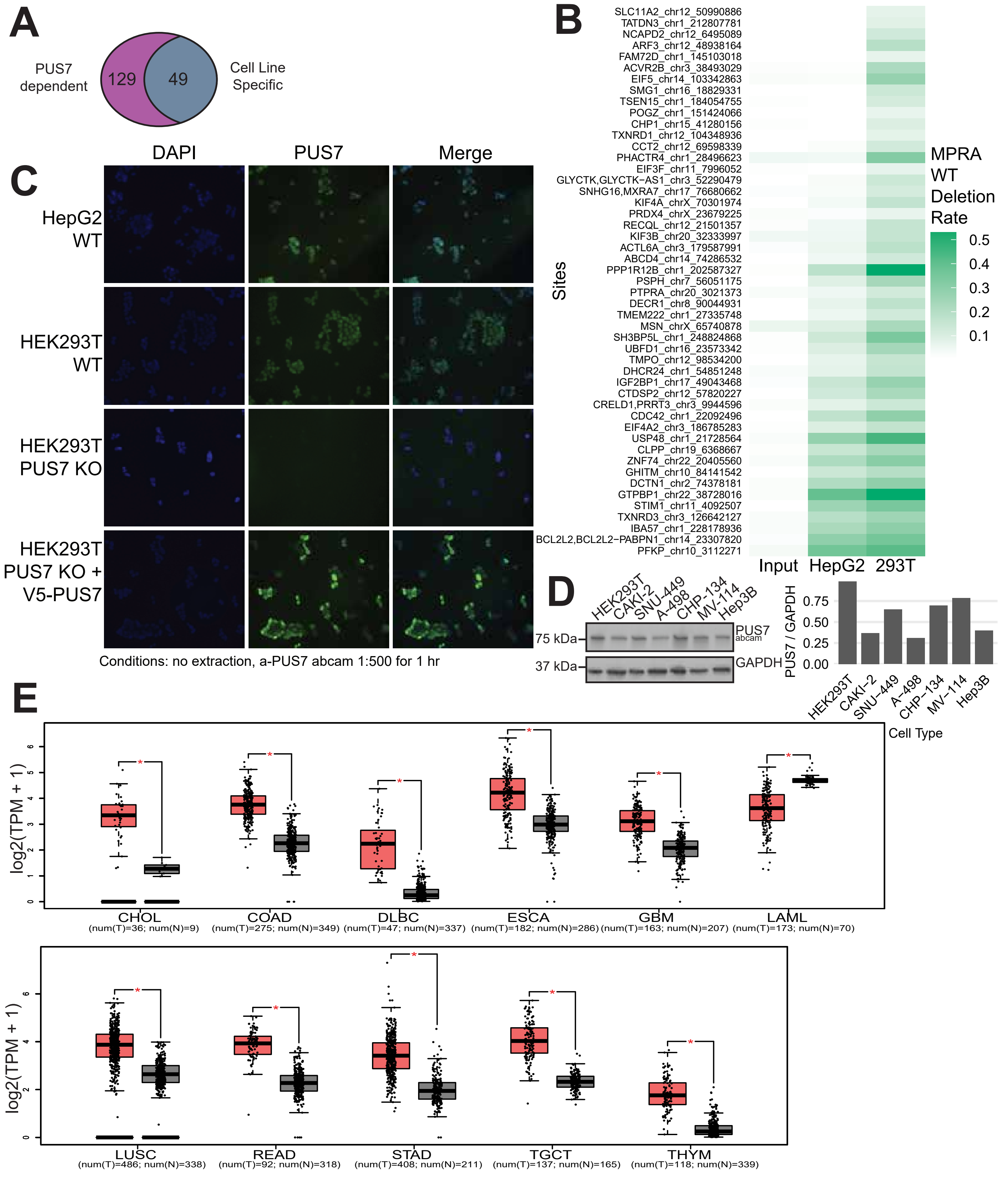

Supplemental Figure 6
